# Supplementary material for: A study on optimization of delayed production mode of iron and steel enterprises based on data mining
Source: PLoS One. 2023 Jan 18;18(1):e0278750. doi: 10.1371/journal.pone.0278750 (PMC9847961; doi:10.1371/journal.pone.0278750)
Supplement: Attachment — Submitted filename: Response to Reviewers.docx [file pone.0278750.s001.docx]

**Response to Reviewers**

**1. Is the manuscript technically sound, and do the data support the conclusions?**

The manuscript must describe a technically sound piece of scientific research with data that supports the conclusions. Experiments must have been conducted rigorously, with appropriate controls, replication, and sample sizes. The conclusions must be drawn appropriately based on the data presented.

Reviewer #1: Yes

Reviewer #2: Partly

Reviewer #3: Yes

**Paper revision and question answer:**

Many thanks for the reviewers' suggestions. According to the suggestions, we have added all the experimental data, these data are shared on the public repository ("Figshare"). According to the requirements of enterprises, the data is converted or encrypted. You can use the following website to get the data.

<https://doi.org/10.6084/m9.figshare.21502425> (The enterprise production and operation data and delayed production control data).

<https://doi.org/10.6084/m9.figshare.21502458> (The costs and production time of the PDP candidate locations of different products).

<https://doi.org/10.6084/m9.figshare.21502467> (The costs and production time of the CODP candidate locations of different products) .

<https://doi.org/10.6084/m9.figshare.21502473> (The order information of different products).

At the same time, we compared three data mining algorithms it this paper.

In addition, we further explains the background, reasons and characteristics of the model construction in this paper, and compares the experimental results of other modeling ideas, making the research results and content of this manuscript more reasonable.

**2. Has the statistical analysis been performed appropriately and rigorously?**

Reviewer #1: Yes

Reviewer #2: N/A

Reviewer #3: Yes

**Paper revision and question answer:**

Thank you very much for the suggestions. We have supplemented the data source and analysis reasons of this paper, and combined with the actual production characteristics of iron and steel enterprises, we have supplemented the reasons for such statistical analysis of manuscripts.

**3. Have the authors made all data underlying the findings in their manuscript fully available?**

The PLOS Data policy requires authors to make all data underlying the findings described in their manuscript fully available without restriction, with rare exception (please refer to the Data Availability Statement in the manuscript PDF file). The data should be provided as part of the manuscript or its supporting information, or deposited to a public repository. For example, in addition to summary statistics, the data points behind means, medians and variance measures should be available. If there are restrictions on publicly sharing data—e.g. participant privacy or use of data from a third party—those must be specified.

Reviewer #1: Yes

Reviewer #2: Yes

Reviewer #3: No

**Paper revision and question answer:**

Many thanks for the reviewers' suggestions. We have supplemented all the data required for data mining and numerical experiment. According to the requirements of enterprises, the data is converted or encrypted.

You can use the following website to get the data.

<https://doi.org/10.6084/m9.figshare.21502425> (The enterprise production and operation data and delayed production control data).

<https://doi.org/10.6084/m9.figshare.21502458> (The costs and production time of the PDP candidate locations of different products).

<https://doi.org/10.6084/m9.figshare.21502467> (The costs and production time of the CODP candidate locations of different products) .

<https://doi.org/10.6084/m9.figshare.21502473> (The order information of different products).

**4. Is the manuscript presented in an intelligible fashion and written in standard English?**

PLOS ONE does not copyedit accepted manuscripts, so the language in submitted articles must be clear, correct, and unambiguous. Any typographical or grammatical errors should be corrected at revision, so please note any specific errors here.

Reviewer #1: Yes

Reviewer #2: Yes

Reviewer #3: Yes

**Paper revision and question answer:**

Thank you very much for the reviewer's encouragement and suggestions. We have optimized the manuscript and corrected grammatical and wording errors.

**5. Review Comments to the Author**

Please use the space provided to explain your answers to the questions above. You may also include additional comments for the author, including concerns about dual publication, research ethics, or publication ethics. (Please upload your review as an attachment if it exceeds 20,000 characters)

**Reviewer #1:**

1. Poor Literature Review: Authors need to include latest and recent research articles.

Response to Reviewer #1's question 1:

(1) Modification description

We have read a lot of the latest relevant literature and updated the references [3], [4], [5], [8], [13], [15], [16], [20], [21], [22], [25], [26], [35]. In addition, in "2.3 Research on delayed production mode in process production enterprises" section, the description of data mining, C-LSTM artificial neural network algorithm and research on delayed production mode is added, focusing on the statistical analysis of the research object and content of delayed production mode.

(2) Revision of manuscript (2.3 Research on delayed production mode in process production enterprises)

According to the literature research, the research of data mining include definitions, technologies, algorithms, tools, fields, scenarios, applications, etc. In the application of data mining, it is concentrated in the fields of medicine, education, management and manufacturing, but the number of the latest literature on the application of manufacturing is few.

The research on the delayed production mode in the literature focused on the supply chain or discrete enterprises. Generally, PDP and CODP were considered separately. A small number of literature considered PDP and CODP at the same time. In addition, the focus of literature research includes CODP location, PDP location, CODP location and PDP location, CODP and inventory management. According to the actual situation of delayed production mode in continuous production enterprises and literature research, this paper is convinced that CODP location, PDP location, and various semi-finished product inventory distribution should be considered simultaneously to achieve effective control of delayed production.

2. Authors need to follow the Journal format for publication.

Modified according to the Journal format for publication.

3. References should be in a proper format as per the journal.

Modified according to the requirements.

4. Reference [3], Author should write the full name of journal.

Reference [3] has been updated and the format have been modified.

5. Reference [24], Author should write the name of journal.

Reference [24] has been updated and the format have been modified.

**Reviewer #2:**

In this paper authors have used the convolutional neural network-long short-term memory artificial neural network algorithm(C-LSTM) in data mining technology to analyze and determine factors that have an impact on delayed production mode in the internal and external production and operation of enterprises.Some of the suggestions are as follows:

1. Discuss the related work in detail. It will help the readers to understand the recent developments. Add papers from 2020, 2021 and 2022. Add a paragraph for the overall review analysis and outcome at the end of the literature.

Response to Reviewer #2's question 1:

(1) Modification description

We went through multiple latest relevant literature and updated the references. In "2.3 Research on delayed production mode in process production enterprises" section, the description of data mining, C-LSTM artificial neural network algorithm and research on delayed production mode is added, focusing on the statistical analysis of the research object and content of delayed production mode.

(2) Revision of manuscript (2.3 Research on delayed production mode in process production enterprises)

According to the literature research, the research of data mining includes definitions, technologies, algorithms, tools, fields, scenarios, applications, etc. In the application of data mining, it is concentrated in the fields of medicine, education, management and manufacturing, but the number of the latest literature on the application of manufacturing is few.

The research on the delayed production mode in the literature focused on the supply chain or discrete enterprises. Generally, PDP and CODP were considered separately. A small number of literature considered PDP and CODP at the same time. In addition, the focus of literature research includes CODP location, PDP location, CODP location and PDP location, CODP and inventory managemet. According to the actual situation of delayed production mode in continuous production enterprises and literature research, this paper proved that CODP location, PDP location, and various semi-finished product inventory distribution should be considered simultaneously to achieve effective control of delayed production.

2. Add numerical outcomes of results in the abstract.

Response to Reviewer #2's question 2:

(1) Modification description

In “Abstract”, the results of PDP-based modeling, CODP-based modeling, PDP and CODP-based modeling, PDP and CODP-based modeling and data mining modeling are compared. The model constructed based on data mining technology in this paper can more accurately describe the situation of delayed production in iron and steel enterprises.

(2) Revision of manuscript (Abstract)

According to the research, in scenarios of considering PDP alone, considering CODP alone, considering both PDP and CODP, considering PDP and CODP and using data mining technology to model, the matching degrees of these methods with the actual situation of the enterprise are 31.8%, 61.4%, 71.6% and 86.6%, respectively.

3. Discuss the corelation between attributes.

Response to Reviewer #2's question 3:

(1) Modification description

In the "5. Discussion" section, the reasons for analyzing and discussing different attributes (model parameters) and the impact of these parameters on the enterprise are added.

(2) Revision of manuscript (5. Discussion)

The main parameters of the model constructed in this paper include: holding cost, return cost, delay penalty cost, production cost, early delivery benefit, customer service level, etc. The decision-making content includes: CODP location, PDP location, CODP corresponding semi-finished product inventory, PDP corresponding semi-finished product inventory.

For the management of delayed production in iron and steel enterprises, customer service level, early delivery benefit, and production delay penalty cost are more important for enterprise operation. Compared with holding cost and return cost, production cost changes more frequently and is more important to the production process of enterprises. Therefore, this paper analyzes the customer service level, delay penalty coefficient, early delivery benefit coefficient and production cost, and studies the impact of their numerical changes on the delayed production control.

4. Provide the comparison with different features.

Response to Reviewer #2's question 4:

(1) Modification description

By our best understanding of this suggestion, we added three supplements.

First, in the "4.3 Model solution" section, I added a comparison of the matching degree between different model scenarios and the actual situation of enterprises.

Second, in the "3.3 Comparison of data mining algorithms" section, we added a comparison of regression errors of C-LSTM artificial neural network algorithm, random forest algorithm, convolutional neural network algorithm and other data mining technologies.

Third, in the "5 Discussion" section, we discusses the impact of different attribute (model parameter) changes on the enterprise.

(2) Revision of manuscript (4.3 Model solution/3.3 Comparison of data mining algorithms/5 Discussion)

Modifications to "4.3 Model solution"

The above results are models constructed based on data mining results. This paper constructed models for considering PDP alone, CODP alone, considering both PDP and CODP. The research results show that, considering PDP alone, CODP alone, considering both PDP and CODP, considering PDP and CODP and using data mining technology to model, the matching degrees of these methods with the actual situation of the enterprise are 31.8%, 61.4%, 71.6% and 86.6%, respectively. So, the model constructed based on data mining technology in this paper can more accurately describe the situations of delayed production in iron and steel enterprises.

Modifications to "3.3 Comparison of data mining algorithms" section.

For data mining task, this paper used the C-LSTM artificial neural network algorithm, random forest algorithm and convolution neural network（CNN） algorithm for calculation. The minimum regression error, maximum regression error and average regression error of these algorithms are shown in Table 3.

Table 3: Regression error comparison of different algorithms

|  | Hot-Rolled Products | | | Chilled Products | | | Galvanized Products | | |
| --- | --- | --- | --- | --- | --- | --- | --- | --- | --- |
|  | Max | Min | Average | Max | Min | Average | Max | Min | Average |
| C-LSTM | 0.45 | 0.008 | 0.1 | 0.34 | 0.011 | 0.085 | 0.34 | 0.009 | 0.075 |
| Random Forest | 0.35 | 0.039 | 0.13 | 0.33 | 0.022 | 0.097 | 0.26 | 0.021 | 0.076 |
| CNN | 0.43 | 0.041 | 0.15 | 0.32 | 0.025 | 0.103 | 0.33 | 0.025 | 0.085 |

Modifications to "5 Discussion" section

According to the above analysis, changes in customer service level and delay penalty coefficient have a gradual and gentle impact on the delayed production control and the total cost. The change of production cost and early delivery benefit coefficient has a gradual and gentle impact on the total cost, but has an abrupt impact on delayed production control. This is because the change of production cost between the CODP and PDP candidate locations is floating, and the production cost increases first and then decreases along the production flow. In addition, the discontinuity of the influence of the early delivery benefit coefficient on the control of delayed production is because in enterprises, the customer service level is usually high, most products will be delivered in advance, and the change of the early delivery benefit coefficient will affect all products produced, resulting in a large cost change from the small change of the early delivery benefit coefficient.

5. The improvement should be specified properly with the reasons, justification and comparative study.

Response to Reviewer #2's question 5:

(1) Modification description

In the "4.1 Question description" section, the research situation of the delayed production mode is described. In combination with the characteristics of iron and steel enterprises, the reason and rationality of this research are explained

(2) Revision of manuscript (4.1 Question description)

Iron and steel enterprises are typical continuous production enterprises. Delayed production will inevitably produce redundant semi-finished products inventory. Some of the inventory is converted into the semi-finished products inventory corresponding to CODP, the rest of the inventory is usually converted into the semi-finished products inventory corresponding to PDP. Therefore, the model should consider PDP, CODP and semi-finished products inventory at the same time. However, relevant factors considered in previous models are mainly by production management experience and financial indicators, these factors belong to the internal factors of the enterprise, and the influence of external factors of the enterprise needs to be obtained and verified through data mining. The above data mining has determined that various supply indexes and comprehensive product prices have strong independent effects on delayed production. Therefore, when building a delayed production model, external factors need to be transformed into relevant parameters. Based on this research idea, the model is constructed as follows.

6. Include proper discussion of the results impact.

Response to Reviewer #2's question 6:

(1) Modification description

In the "6. Conclusions" section, the impact on research in this field is introduced.

(2) Revision of manuscript (6. Conclusions)

This research is to help enterprises use data mining technology to more effectively consider various internal and external factors on delayed production, and then effectively optimize the model.

7. What are the limitations of the approach presented?

Response to Reviewer #2's question 7:

(1) Modification description

In the "6. Conclusions" section, we improved discussion of research limitations.

(2) Revision of manuscript (6. Conclusions)

This study has limitations, mainly in three aspects: First, the model does not consider the impact of multi cycle, and some continuous production enterprises may adjust the location of PDP or CODP in a short time, so it is necessary to consider the situation of multi cycle; Second, the C-LSTM artificial neural network algorithm used in this paper may not be applicable to all types of data when processing data inside and outside the enterprise, so more data mining methods should be considered; Third, the data collected in this paper is incomplete, so the results of relevant analysis may need to be further verified.

8. Image quality should be improved.

All pictures have been redrawn and replaced, and the image quality has been improved, including Figure 2, Figure 3, Figure 4, Figure 5, Figure 6, Figure 7, Figure 9, Figure 10, Figure 11, Figure 12, Figure 13, Figure 14, Figure 15, and Figure 16.

9. How the results can be replicated in the future?

Response to Reviewer #2's question 9:

(1) Modification description

In the "6. Conclusions" section, the practical significance of this model are introduced and the replicated of the model is explained.

(2) Revision of manuscript (6. Conclusions)

Combined with the model constructed and the experimental data in this paper, the research results of can be replicated and verified, and this research method for delayed production mode in continuous production enterprises can be reused. Its research methods and modeling ideas have quantitative practicability and can be directly applied to the delayed production process in continuous production enterprises, including oil refineries, chemical plants, glass processing plants, food processing plants, etc.

10. Discuss the impact of external factors.

Response to Reviewer #2's question 10:

(1) Modification description

The influence of external factors was obtained through data mining and transformed into model parameters, namely early delivery coefficient and product cost, which are discussed in "5. Discussion" section.

(2) Revision of manuscript (5. Discussion)

Therefore, when building a delayed production model, external factors need to be transformed into relevant parameters. Based on this research idea, the model is constructed as follows.

**Reviewer #3:**

1. Abstract :- Is there any significant improvement in accuracy between experiment with conventional and proposed method with respect to CODP and PDP? Please give improvement result in percentage.

Response to Reviewer #3's question 1:

(1) Modification description

In the conventional research of delayed production mode, most literature consider PDP alone or consider CODP alone, a few literature consider PDP and CODP at the same time. This paper paper consider PDP and CODP at the same time, and use data mining technology. So, in “Abstract”, the results of PDP-based modeling, CODP-based modeling, PDP and CODP-based modeling, PDP and CODP-based modeling and data mining modeling are compared.

(2) Revision of manuscript (Abstract)

According to the research, in scenarios of considering PDP alone, considering CODP alone, considering both PDP and CODP, considering PDP and CODP and using data mining technology to model, the matching degree of these methods with the actual situation of the enterprise is 31.8%, 61.4%, 71.6% and 86.6%, respectively.

2. Dataset:- Data set analysis is not cleared understandable ? What is the size of data set and where is the link from which you obtained without any restriction?

Response to Reviewer #3's question 2:

(1) Modification description

In "3.2 Analysis of data mining results" section, the data and information of data mining in this paper are supplemented, these data are shared on the public repository ("Figshare"). According to the requirements of enterprises, the data is converted or encrypted. You can use the following website to get the data.

<https://doi.org/10.6084/m9.figshare.21502425> (The enterprise production and operation data and delayed production control data).

<https://doi.org/10.6084/m9.figshare.21502458> (The costs and production time of the PDP candidate locations of different products).

<https://doi.org/10.6084/m9.figshare.21502467> (The costs and production time of the CODP candidate locations of different products) .

<https://doi.org/10.6084/m9.figshare.21502473> (The order information of different products).

(2) Revision of manuscript (3.2 Analysis of data mining results)

This paper used the C-LSTM artificial neural network algorithm, PyCharm as integrated development environment, and Python 3.9 for programming, and analyzed production and operation data and delay control data.

All data are from a large iron and steel enterprise with good operation or information platform. The data collection scope is 5 years, and the time unit is weekly, including 17 market factors, 8 business factors, 11 production factors, and 12 delayed production control data. According to the confidentiality requirements of the enterprise, data were encrypted without affecting the analysis results. The data used for data mining analysis is "The enterprise production and operation data and delayed production control data". These data are shared on the public repository ("Figshare"). You can find the sharing link in the "Data availability statement". The results of the analysis are as follows.

3. In figure 7, it has not been cleared the error loss by C-LSTM and Random forest with respect to the chilled ,hot-rolled and galvanized products.

Response to Reviewer #3's question 3:

(1) Modification description

In“3.3 Comparison of data mining algorithms”section, figure 7 is redrawn, and illustrates the three products in Figure 7.

(2) Revision of manuscript (3.3 Comparison of data mining algorithms)

The regression errors of C-LSTM artificial neural network algorithm and random forest algorithm are analyzed in detail, and the results are shown in Figure 7.

Figure 7: Regression error comparison of C-LSTM artificial neural network algorithm and random forest algorithm

As shown in Figure 7, the horizontal axis (x-axis) represents the regression error of hot-rolled products, the longitude axis (y-axis) represents the regression error of chilled products, and the vertical axis (z-axis) represents the regression error of galvanized products. The blue dot indicates the result of using the C-LSTM artificial neural network algorithm, and the red dot indicates the result of using the random forest algorithm. The blue dots are concentrated and closer to the origin, while the red dots are scattered and far away from the origin. Therefore, the comparative analysis of the algorithms indicated that the regression error of the C-LSTM artificial neural network algorithm was smaller, and thus more effective.

4. Please provide the error loss in percentages for both proposed model and state of art models in table form for figure 7.

Response to Reviewer #3's question 4:

(1) Modification description

In the "3.3 Comparison of data mining algorithms" section , regression errors comparison of C-LSTM artificial neural network algorithm, random forest algorithm and convolutional neural network algorithm were added, and Table 3 was drawn to show.

(2) Revision of manuscript (3.3 Comparison of data mining algorithms)

For data mining task, this paper used the C-LSTM artificial neural network algorithm, random forest algorithm and convolution neural network（CNN） algorithm for calculation. The minimum regression error, maximum regression error and average regression error of these algorithms are shown in Table 3.

Table 3: Regression error comparison of different algorithms

|  | Hot-Rolled Products | | | Chilled Products | | | Galvanized Products | | |
| --- | --- | --- | --- | --- | --- | --- | --- | --- | --- |
|  | Max | Min | Average | Max | Min | Average | Max | Min | Average |
| C-LSTM | 0.45 | 0.008 | 0.1 | 0.34 | 0.011 | 0.085 | 0.34 | 0.009 | 0.075 |
| Random Forest | 0.35 | 0.039 | 0.13 | 0.33 | 0.022 | 0.097 | 0.26 | 0.021 | 0.076 |
| CNN | 0.43 | 0.041 | 0.15 | 0.32 | 0.025 | 0.103 | 0.33 | 0.025 | 0.085 |

5.In model construction 4.2, It is not cleared how model generate the optimization results as it should have compared the existing optimization results!

Response to Reviewer #3's question 5:

(1) Modification description

In the "4.1 Question description" section, we added the reason why we should adopt this idea to build the model. At the same time, in the "4.2 Model construction" section, we added the solution method and software introduction of the model, and compared the matching degree between different modeling ideas and the actual situation of the enterprise

(2) Revision of manuscript (4.1 Question description/4.2 Model construction)

Modifications to "4.1 Question description"

Iron and steel enterprises are typical continuous production enterprises. Delayed production will inevitably produce redundant semi-finished products inventory. Some of the inventory is converted into the semi-finished products inventory corresponding to CODP, the rest of the inventory is usually converted into the semi-finished products inventory corresponding to PDP. Therefore, the constructed model should consider PDP, CODP and semi-finished products inventory at the same time. However, relevant factors considered in previous models are mainly based on production management experience and financial indicators, these factors belong to the internal factors of the enterprise, and the influence of external factors of the enterprise needs to be obtained and verified through data mining. The above data mining has determined that various supply indexes and comprehensive product prices have strong independent effects on delayed production. Therefore, when building a delayed production model, external factors need to be transformed into relevant parameters. Based on this research idea, the model is constructed.

Modifications to "4.2 Model construction"

Other information includes product category PDP candidate location information, product CODP candidate location information, product order information, etc. These data are encrypted according to the requirements of the enterprise. The data used for model solution include "The costs and production time of the PDP candidate locations of different products", "The costs and production time of the CODP candidate locations of different products" and "The order information of different products". These data are shared on the public repository ("Figshare"). You can find the sharing link in the "Data availability statement".

The model constructed in this paper is nonlinear mixed integer programming model, which is solved by using a precise algorithm and realized by lingo software. The optimal decision is as follows.

The above results are models constructed based on data mining results. This paper constructed models for considering PDP alone, CODP alone, considering both PDP and CODP. The research results show that, considering PDP alone, CODP alone, considering both PDP and CODP, considering PDP and CODP and using data mining technology to model, the matching degrees of these methods with the actual situation of the enterprise are 31.8%, 61.4%, 71.6% and 86.6%, respectively. So, the model constructed based on data mining technology in this paper can more accurately describe the situations of delayed production in iron and steel enterprises.

6. In Working of Proposed model i.e combination of CNN and LSTM should be more precisely and apparent.

Response to Reviewer #3's question 6:

(1) Modification description

In the "3.1 Task description of data mining" section, the feature description and implementation process of C-LSTM artificial neural network algorithm are added.

(2) Revision of manuscript (3.1 Task description of data mining)

The main task of data mining in this paper is to use the technology of C-LSTM artificial neural network algorithm, which is to process the data with business relationships and time sequences. In the front part, a one-dimensional convolution layer is used to extract the business relationships, and the intrinsic coupling characteristics of each signal are extracted by reducing sampling and filtering. In the back part, long-time and short-memory layer is used to extract the time sequences, thus realizing the function of fast and effective data extraction. The analysis results are shown as follows.

**6. PLOS authors have the option to publish the peer review history of their article (what does this mean?). If published, this will include your full peer review and any attached files.**

If you choose “no”, your identity will remain anonymous but your review may still be made public.

Reviewer #1: No

Reviewer #2: No

Reviewer #3: No

[NOTE: If reviewer comments were submitted as an attachment file, they will be attached to this email and accessible via the submission site. Please log into your account, locate the manuscript record, and check for the action link "View Attachments". If this link does not appear, there are no attachment files.]

While revising your submission, please upload your figure files to the Preflight Analysis and Conversion Engine (PACE) digital diagnostic tool, https://pacev2.apexcovantage.com/. PACE helps ensure that figures meet PLOS requirements. To use PACE, you must first register as a user. Registration is free. Then, login and navigate to the UPLOAD tab, where you will find detailed instructions on how to use the tool. If you encounter any issues or have any questions when using PACE, please email PLOS at figures@plos.org. Please note that Supporting Information files do not need this step.

**7. Other modification description**

(1) The data that support the findings of this study are openly shared on the public repository ("Figshare").

(2) If possible, we would like to modify the information of the author, and we have written a letter. Please refer to Attachment.

(3) We have used the PACE software to modify all images and upload them.

(4) The title of this paper were revised.

Original title: "Optimization of delayed production mode of iron and steel enterprises based on data mining technology"

Current title: "A Study on optimization of delayed production mode of iron and steel enterprises based on data mining"

(5) The title of 3.0 sections were revised.

Original title: "3.Using data mining technology to analyze the influencing factors of delayed production"

Current title: "3. Data mining to analyze the influencing factors of delayed production"

(6) The title of 3.2 sections were revised.

Original title: "3.2 Analysis of data mining results"

Current title: "3.2 Analysis of data mining"
